# Effect of abaloparatide on vertebral, nonvertebral, major osteoporotic, and clinical fractures in a subset of postmenopausal women at increased risk of fracture by FRAX probability

**DOI:** 10.1007/s11657-019-0564-7

**Published:** 2019-02-05

**Authors:** E. V. McCloskey, L. A. Fitzpatrick, M.-Y. Hu, G. Williams, J. A. Kanis

**Affiliations:** 1 Centre for Metabolic Bone Diseases, University of Sheffield, Sheffield, UK; 2 Metabolic Bone Centre, Sorby Wing, Northern General Hospital, Herries Road, Sheffield, S57AU UK; 3 Radius Health, Inc., Waltham, MA USA

**Keywords:** Abaloparatide, FRAX, High risk, Fracture, Osteoporosis

## Abstract

**Summary:**

We evaluated the efficacy of abaloparatide in women who were at increased risk for fracture, based on CHMP recommended risk thresholds, at the Abaloparatide Comparator Trial In Vertebral Endpoints (ACTIVE) study baseline. Among patients at high risk based on FRAX probabilities, 18 months of abaloparatide significantly decreased risk for all fracture endpoints compared with placebo.

**Purpose:**

Abaloparatide, a novel anabolic agent for the treatment of postmenopausal osteoporosis, significantly reduced the risk of vertebral and nonvertebral fractures in the ACTIVE study compared with placebo. In this post hoc analysis, we evaluated abaloparatide’s efficacy in a subset of women in the study at an increased risk of fracture at baseline, based on the Committee for Medicinal Products for Human Use (CHMP) recommended risk thresholds for inclusion in clinical trials.

**Methods:**

Women with a baseline 10-year risk of major osteoporotic fracture ≥ 10% or hip fracture ≥ 5%, assessed using the FRAX® tool (including femoral neck bone mineral density), were included in the analysis. The proportion with one or more events of new morphometric vertebral fractures was calculated. Event rates for nonvertebral, major osteoporotic, and all clinical fractures were estimated using Kaplan-Meier analysis.

**Results:**

Following 18 months of treatment, abaloparatide significantly reduced incident vertebral fractures compared with placebo (relative risk reduction = 91%; 0.5% versus 5.6%; *p* < 0.001). Abaloparatide treatment was also associated with significantly fewer nonvertebral, major osteoporotic, and clinical fractures compared with placebo: 2.7% versus 5.8%, *p* = 0.036; 1.3% versus 6.0%, *p* < 0.001; and 3.5% versus 8.2%, *p* = 0.006, respectively. The effect of abaloparatide on major osteoporotic fractures (78% reduction) was significantly greater than that seen with teriparatide (23% reduction, *p* = 0.007).

**Conclusion:**

In a subset of postmenopausal women at increased risk of fracture as judged by CHMP guidance, abaloparatide significantly decreased the risk of all fracture endpoints compared with placebo.

## Introduction

Osteoporosis is still undertreated despite many years of advances, particularly in its diagnosis, assessment of fracture risk, and development of interventions that reduce the risk of fractures [1]. There are several classes of approved osteoporosis therapies, which either inhibit osteoclast-mediated bone resorption (bisphosphonates, selective estrogen receptor modulators, and the monoclonal antibody to receptor activator of nuclear kappa-B ligand) or stimulate osteoblast-mediated bone formation (teriparatide and abaloparatide) [2].

Abaloparatide is a 34-amino acid synthetic analog of the human parathyroid-related protein (PTHrP) and is a selective activator of the PTH type 1 receptor (PTHR1) signaling pathway. Abaloparatide has higher affinity for the RG vs R^0^ conformation of the PTHR1, resulting in more transient receptor signaling consistent with a net anabolic effect [3, 4].

The Abaloparatide Comparator Trial In Vertebral Endpoints (ACTIVE, NCT01343004) was an international, randomized, placebo- and active-controlled phase 3 trial including postmenopausal women with osteoporosis [5]. The primary objective of ACTIVE was to determine the efficacy and adverse events of subcutaneous (SC) abaloparatide compared with placebo for prevention of new morphometric vertebral fractures. Treatment with abaloparatide significantly reduced the risk of new vertebral (*p* < 0.001) and nonvertebral fractures (*p* = 0.049) compared with placebo, and the risk of major osteoporotic fractures compared with placebo (*p* < 0.001) and with teriparatide (*p* = 0.03) over 18 months [5].

The absolute risk of fracture depends on age, life expectancy, and the current relative risk. The International Osteoporosis Foundation and World Health Organization recommend that risk of fracture be expressed as a short-term absolute risk, i.e., probability over a 10-year interval as given, for example, by FRAX® [6, 7]. Against this background, the Committee for Medicinal Products for Human Use (CHMP) revised its guidelines on the evaluation of medicinal products in the treatment of primary osteoporosis [8], which came into effect at the end of May 2007. A major departure from previous guidance is that there is no longer any distinction between prevention and treatment, but there is an emphasis on the study of patients at high risk of fracture. The preferred metric for expressing risk is the 10-year probability of fracture. Suggested probabilities as inclusion criteria are 15 to 20% for spine fracture, 5 to 7.5% for hip fracture, and 10 to 15% for major nonvertebral fractures [8].

FRAX is a fracture risk assessment tool based on the use of clinical risk factors with or without bone mineral density (BMD) testing. The FRAX models provide a 10-year probability of hip fracture and a 10-year probability of major osteoporotic fracture [9]. Several studies have shown that patients across a range of fracture risk as assessed by FRAX algorithms benefit from substantial reductions in fracture risk with bone-targeted therapies [10–14].

The primary objective of the present analysis was to determine the efficacy of abaloparatide in a subset of women in ACTIVE who were at increased risk of fracture at baseline determined by FRAX and consistent with the CHMP thresholds.

## Methods

The design and methods of ACTIVE have been described in detail elsewhere [5]. A total of 2463 postmenopausal women with osteoporosis, defined as a T score of ≤ − 2.5 and > − 5.0 or ≤ − 2.0 and > − 5.0 (aged > 65 years) at the lumbar spine or femoral neck, together with radiologic evidence of at least 1 prior vertebral fracture at any time or a history of prior nonvertebral fracture within the last 5 years, were randomized to receive double-blind abaloparatide 80 μg/d (Tymlos; Radius, Waltham, MA) or placebo, or open-label teriparatide 20 μg/d administered SC for 18 months. For the purposes of the primary efficacy endpoint (1 or more new morphometric vertebral fractures), a modified intent-to-treat (mITT) population, which included all ITT randomized patients who had a pretreatment and a postbaseline evaluable radiologic assessment, was evaluated. All other fracture endpoints were evaluated in the ITT population. Morphometric vertebral fractures were assessed on anteroposterior and lateral radiographs of the lumbar and thoracic spine obtained at baseline and the end of treatment, using the semiquantitative technique of Genant, while nonvertebral fractures were initially self-reported but required verification from source documents. Treatment groups were blinded from all assessors [5].

The post hoc subgroup analysis reported here was restricted to patients with a baseline 10-year probability of major osteoporotic fracture of ≥ 10% or hip fracture of ≥ 5%, as calculated by FRAX with BMD. The calculation of baseline FRAX probability was undertaken independently using the usual FRAX clinical risk factors alone or with femoral neck BMD, and both outputs were calculated and reported [12].

## Statistical analysis

The proportion of patients with 1 or more incident morphometric vertebral fractures was calculated. Other fracture types evaluated included nonvertebral fractures, clinical fractures (all fractures that would cause a patient to seek medical care, regardless of the level of trauma, including clinical spine), and major osteoporotic fractures (fractures of the upper arm, wrist, hip, or clinical spine). Nonvertebral fractures excluded those of the spine, sternum, patella, toes, fingers, skull, and face and those with high trauma, defined as a fall from a height equal to or higher than the level of a stool, chair, or the first rung of a ladder). The percentage of new vertebral fractures was calculated using the mITT population at 18 months. The percentage of nonvertebral, clinical, and major osteoporotic fractures was estimated using the Kaplan-Meier method using the ITT population at 19 months (the entire observational period including 18 months of treatment plus 1 month of follow-up).

For treatment group comparisons, the percentage of new vertebral fractures at 18 months was compared using the Fisher’s exact test, and the 95% CI of the relative risk reduction was derived using the Wald’s method [15]. Times to first nonvertebral, major osteoporotic, and clinical fractures through the 19 months of study period were summarized using the Kaplan-Meier method and were compared using the log-rank test. Hazard ratios (HR) and 95% CIs were derived using the Cox proportional hazards model.

## Results

Of the 2463 women in ACTIVE, 1400 (56.8%) were at increased risk of fracture by CHMP thresholds and included in this analysis. This comprised 468 women in the placebo group, 459 in the abaloparatide group, and 473 in the teriparatide group with a mean baseline 10-year probability of major osteoporotic fracture of 17.9%, 18.2%, and 17.9%, respectively. The baseline characteristics of all three treatment groups were well matched (Table 1); specifically, there were no significant differences in FRAX fracture probabilities (calculated with or without BMD) across the groups. For new vertebral fracture outcomes, the mITT populations comprised 414, 401, and 430 patients in the placebo, abaloparatide, and teriparatide groups, respectively. As with the ITT populations, there were no significant differences in baseline characteristics including FRAX fracture probabilities (calculated with or without BMD) across the groups.

**Table 1:** Baseline characteristics of ACTIVE study patients with increased risk^a^ of fracture by FRAX (ITT population)

	Placebo (*n* = 468)	Abaloparatide (*n* = 459)	Teriparatide (*n* = 473)
Age, years, mean (SD)	70.0 (6.27)	69.9 (6.67)	69.9 (6.37)
Race, *n* (%)			
White	354 (75.6)	352 (76.7)	356 (75.3)
Asian	108 (23.1)	102 (22.2)	109 (23.0)
Black or African American	2 (0.4)	4 (0.9)	4 (0.8)
Other	4 (0.9)	1 (0.2)	4 (0.8)
BMI, kg/m^2^, mean (SD)	24.70 (3.487)	24.48 (3.340)	24.86 (3.540)
Lumbar spine BMD T-score, mean (SD)	− 2.89 (0.854)	− 2.79 (0.939)	− 2.80 (0.937)
Total hip BMD T-score, mean (SD)	− 2.18 (0.700)	− 2.12 (0.680)	− 2.09 (0.700)
Femoral neck BMD T-score, mean (SD)	− 2.42 (0.604)	− 2.40 (0.576)	− 2.38 (0.624)
10-year probability of MOF calculated with BMD, %, mean (SD)	17.94 (6.88)	18.23 (7.59)	17.89 (7.36)
10-year probability of hip fracture calculated with BMD, %, mean (SD)	6.92 (4.69)	7.20 (5.65)	7.10 (5.34)

^a^10-year probability of MOF of ≥ 10% or hip fracture of ≥ 5% as calculated by FRAX

*BMD* bone mineral density, *ITT* intent to treat, *MOF* major osteoporotic fracture, *SD* standard deviation

**Fig. 1:**
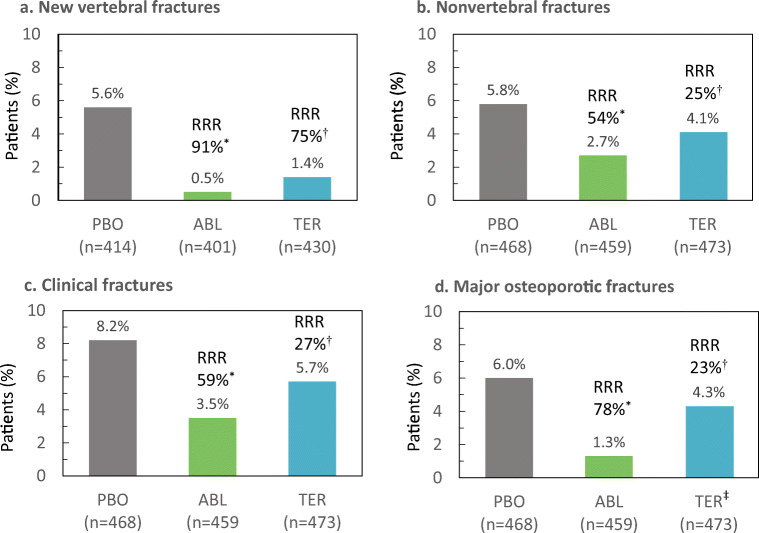
Fracture rates and RRR for new vertebral fractures (**a**), nonvertebral fractures (**b**), clinical fractures (**c**), and major osteoporotic fractures (**d**) following treatment with placebo (PBO), abaloparatide (ABL), or teriparatide (TER) in patients at increased risk of fracture by FRAX **a** ^*^p < 0.001 abaloparatide versus placebo; ^†^*p* = 0.001 teriparatide versus placebo. **b** ^*^p = 0.036 abaloparatide versus placebo; ^†^nonsignificant teriparatide versus placebo. **c** ^*^p < 0.001 abaloparatide versus placebo; ^†^nonsignificant teriparatide versus placebo; ^‡^*p* = 0.007 abaloparatide versus teriparatide. **d** ^*^p = 0.006 abaloparatide versus placebo; ^†^nonsignificant teriparatide versus placebo. Event rates in **b**, **c**, and **d** are Kaplan-Meier estimates at 19 months (18 months active treatment + 1-month follow-up). RRR relative risk reduction

Results for the primary efficacy endpoint in this subgroup of patients at increased risk of fracture are shown in Fig. 1. The proportions of patients with new morphometric vertebral fractures were 0.5% (*n* = 2) in the abaloparatide group and 5.6% (*n* = 23) in the placebo group, representing a 91% reduction in the risk of fracture in the abaloparatide group compared with the placebo group (*p* < 0.001; Fig. 1a). New morphometric vertebral fractures occurred in 1.4% (*n* = 6) of the patients in the teriparatide group, representing a 75% fracture risk reduction compared with the placebo (*p* = 0.001). The difference in reduction between abaloparatide and teriparatide was not statistically significant.

Kaplan-Meier estimated event rates for nonvertebral fractures were 2.7% (*n* = 10) in the abaloparatide group and 5.8% (*n* = 23) in the placebo group, representing a 54% reduction in the risk of fracture with abaloparatide versus placebo (*p* = 0.036; Fig. 1b). The estimated event rate for nonvertebral fractures in the teriparatide group (4.1%, *n* = 18) was not significantly different from that with placebo or abaloparatide (*p* = 0.365 and *p* = 0.202, respectively).

Kaplan-Meier estimated event rates for major osteoporotic fractures are presented in Fig. 1c, with rates of 1.3% (*n* = 5) in the abaloparatide group and 6.0% (*n* = 24) in the placebo group, representing a 78% reduction in the risk of major osteoporotic fracture with abaloparatide versus placebo (*p* < 0.001). In the teriparatide group, the estimated event rate for major osteoporotic fractures was 4.3% (*n* = 19), a 23% reduction compared with placebo, which was not statistically significant (*p* = 0.384). The reduction in risk of major osteoporotic fracture with abaloparatide relative to teriparatide was 72% (*p* = 0.007).

Kaplan-Meier estimated event rates for clinical fractures were 3.5% (*n* = 13) in the abaloparatide group and 8.2% (*n* = 33) in the placebo group, representing a 59% reduction in the risk of fracture with abaloparatide versus placebo (*p* = 0.006; Fig. 1d. In the teriparatide group, the estimated event rate was 5.7% (*n* = 25); the difference in the risk of fracture versus placebo or abaloparatide was not significant (*p* = 0.234 and *p* = 0.088, respectively).

**Fig. 2:**
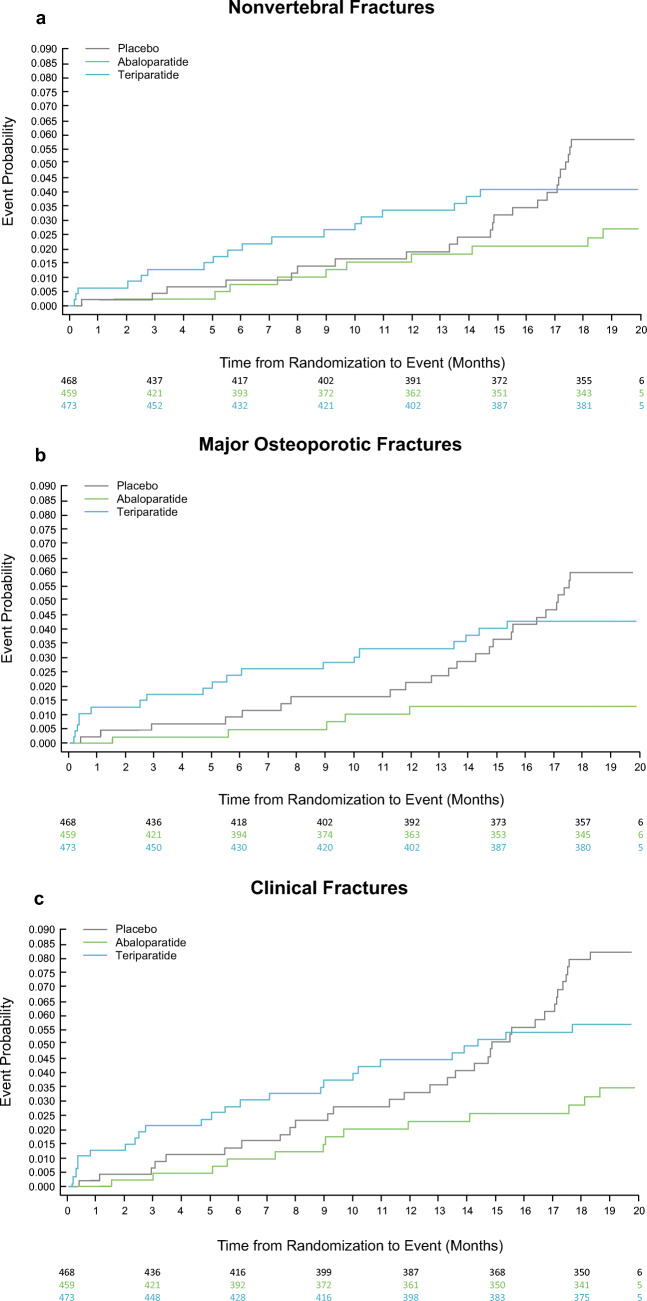
**a** Kaplan-Meier curve of time to first incidence of nonvertebral fracture by treatment group in patients at increased risk of fracture by FRAX. *p* = 0.036, abaloparatide versus placebo. **b** Kaplan-Meier curve of time to first incidence of major osteoporotic fracture by treatment group in patients at increased risk of fracture by FRAX. *p* < 0.001, abaloparatide versus placebo. **c** Kaplan-Meier curve of time to first incidence of clinical fracture by treatment group in patients at increased risk of fracture by FRAX. *p* = 0.006, abaloparatide versus placebo

Kaplan-Meier plots of the time to first incidence of nonvertebral fractures, major osteoporotic fractures, and clinical fractures by treatment group are shown in Fig. 2a, b, and c, respectively.

## Discussion

Our findings demonstrate that in osteoporotic women at increased risk of fracture, consistent with CHMP recommendations for trial inclusion, abaloparatide is associated with a significant reduction in the risk of fracture compared with placebo for new vertebral, nonvertebral, major osteoporotic, and clinical fractures and with a significant reduction in risk of major osteoporotic fracture compared with teriparatide. The reductions in fracture risk with abaloparatide in this analysis are consistent with those reported in previous analyses of prespecified risk subgroups [16] and in the full ACTIVE population for all fractures assessed (Table 2) [5]. In ACTIVE, abaloparatide significantly reduced the risk of new vertebral fractures by 86% compared with placebo (*p* < 0.001), of nonvertebral fractures by 43% compared with placebo (*p* = 0.049), of major osteoporotic fractures by 70% compared with placebo (*p* < 0.001) and by 55% compared with teriparatide (*p* = 0.03), and of clinical fractures by 43% compared with placebo (*p =* 0.02). Indeed, abaloparatide has previously been shown to reduce fracture risk to a similar extent across a wide range of fracture probabilities [12].

**Table 2:** Fracture efficacy in the ACTIVE study and subgroup of patients at increased risk^a^ of fracture by FRAX

	ACTIVE study			Increased risk of fracture by FRAX^a^		
	Abaloparatide versus placebo [5]			Abaloparatide versus placebo		
Fracture event	HR^b^	95% CI	*p* value	HR^b^	95% CI	*p* value
New vertebral	RR, 0.14	0.05–0.39	< 0.001	RR, 0.09	0.02–0.38	< 0.001
Nonvertebral	0.57	0.32–1.00	0.049	0.46	0.22–0.97	0.036
Major osteoporotic	0.30	0.15–0.61	< 0.001	0.22	0.08–0.58	< 0.001
Clinical	0.57	0.35–0.91	0.02	0.41	0.22–0.79	0.006

^a^10-year probability of MOF of ≥ 10% or hip fracture of ≥ 5% as calculated by FRAX

^b^Values are reported as HR (unadjusted) unless otherwise indicated

*CI* confidence interval, *HR* hazard ratio, *RR* relative risk

The effect of teriparatide on vertebral and nonvertebral fracture risk by baseline FRAX fracture probability in a pivotal phase 3 study of 1637 postmenopausal women with osteoporosis has been reported [10]. The study concluded that teriparatide significantly decreased the risk of morphometric vertebral fractures (relative risk reduction [RRR] 66% [95% CI 50–77%]) and all nonvertebral fractures (RRR 37% [95% CI 10–56%]) compared with placebo in women irrespective of baseline fracture probability. In our analysis, teriparatide was associated with similar magnitudes of reductions in the risk of new vertebral and nonvertebral fractures compared with placebo, but the effect on nonvertebral fractures was not statistically significant.

The present analysis has several strengths and limitations. It was conducted within a randomized controlled trial (RCT) where overall efficacy of abaloparatide was previously demonstrated, thus enabling the examination of subgroups to determine benefit. The analysis is in keeping with a previous study of abaloparatide efficacy in the ACTIVE study, where FRAX probability was handled as a continuous variable [12]; this suggested that the efficacy of abaloparatide, when expressed as an HR, was similar across the full spectrum of risk included in the study. A further strength is that the analysis contains an assessment of efficacy of an active comparator (open-label teriparatide) in this group of high-risk patients. Although these findings come from within the setting of an RCT, the main limitation of this post hoc analysis is the relatively low fracture-event rates. Caution needs to be exercised in the interpretation of possible differences between teriparatide and abaloparatide in their effects on major osteoporotic fracture. As noted elsewhere, the difference appears to be driven by a more rapid occurrence of early fractures in women randomized to teriparatide [17], with a lower rate thereafter. The reasons for this remain unclear as it could represent a real effect or a chance occurrence; intriguingly, in the pivotal fracture trial for teriparatide, a similar early excess of fracture events was observed in Kaplan-Meier plots of incident nonvertebral fractures, though the overall effect was to reduce such fractures [18].

## Conclusions

This ACTIVE study subgroup analysis demonstrated that in patients at increased baseline risk of fracture, according to CHMP guidance, abaloparatide significantly decreased the risk of fracture compared with placebo for all endpoints assessed.
